# LungBEAM: A prospective multicenter study to monitor stage IV NSCLC patients with EGFR mutations using BEAMing technology

**DOI:** 10.1002/cam4.4135

**Published:** 2021-07-23

**Authors:** Pilar Garrido, Luis Paz‐Ares, Margarita Majem, Teresa Morán, José Manuel Trigo, Joaquim Bosch‐Barrera, Rosario Garcίa‐Campelo, José Luis González‐Larriba, José Miguel Sánchez‐Torres, Dolores Isla, Núria Viñolas, Carlos Camps, Amelia Insa, Óscar Juan, Bartomeu Massuti, Alfredo Paredes, Ángel Artal, Marta López‐Brea, José Palacios, Enriqueta Felip

**Affiliations:** ^1^ Medical Oncology Department IRYCIS Hospital Universitario Ramón y Cajal Universidad Alcalá Madrid Spain; ^2^ CIBERONC Madrid Spain; ^3^ Medical Oncology Department Hospital Universitario 12 de Octubre and i+12 Research Institute Madrid Spain; ^4^ Lung Cancer Group, Clinical Research Program Spanish National Cancer Research Center (CNIO) Madrid Spain; ^5^ Complutense University Madrid Spain; ^6^ Medical Oncology Department Hospital De La Santa Creu I Sant Pau Barcelona Spain; ^7^ Spanish Lung Cancer Group (GECP) Barcelona Spain; ^8^ ICO Badalona Hospital Germans Trias i Pujol Barcelona Spain; ^9^ Medical Oncology Department Hospital Universitario Virgen de la Victoria Málaga Spain; ^10^ Medical Oncology Catalan Institute of Oncology (ICO), Dr. Josep Trueta Hospital of Girona Girona Spain; ^11^ Medical Oncology Department Hospital Universitario Da Coruña A Coruña Spain; ^12^ Medical Oncology Department Hospital Universitario Clínico San Carlos Madrid Spain; ^13^ Medical Oncology Department Hospital de la Princesa Madrid Spain; ^14^ Medical Oncology Department Hospital Universitario Lozano Blesa Zaragoza Spain; ^15^ Medical Oncology Department Hospital Clinic i Provincial Barcelona Spain; ^16^ Medical Oncology Department, Hospital General Universitario de Valencia Universidad de Valencia Valencia Spain; ^17^ Medical Oncology Department Hospital Clínico Universitario Valencia Spain; ^18^ Medical Oncology Department Hospital Universitario La Fe Valencia Spain; ^19^ Medical Oncology Department Hospital General Universitario de Alicante Alicante Spain; ^20^ Medical Oncology Department Hospital Universitario Donostia Donostia‐San Sebastián Spain; ^21^ Medical Oncology Hospital Universitario Miguel Servet Zaragoza Spain; ^22^ Medical Oncology Department Hospital Universitario Marqués de Valdecilla Santander Spain; ^23^ Pathology Department, IRYCIS Hospital Universitario Ramón y Cajal Universidad Alcalá Madrid Spain; ^24^ Medical Oncology Department Hospital Universitario Vall d’Hebron Barcelona Spain

**Keywords:** BEAMing, *EGFR* mutations, liquid biopsy, non‐small cell lung carcinoma

## Abstract

**Objectives:**

The aim of LungBEAM was to determine the value of a novel epidermal growth factor receptor (*EGFR*) mutation test in blood based on BEAMing technology to predict disease progression in advanced non‐small cell lung cancer (NSCLC) patients treated with first‐ or second‐generation EGFR‐tyrosine kinase inhibitors (EGFR‐TKIs). Another goal was to monitor the dynamics of *EGFR* mutations, as well as to track *EGFR* exon 20 p.T790M (p.T790M) resistance during treatment, as critical indicators of therapeutic efficacy and patient survival.

**Methods:**

Stage IV NSCLC patients with locally confirmed EGFR‐TKI sensitizing mutations (ex19del and/or L858R) in biopsy tissue who were candidates to receive first‐ or second‐generation EGFR‐TKI as first‐line therapy were included. Plasma samples were obtained at baseline and every 4 weeks during treatment until a progression‐free survival (PFS) event or until study completion (72‐week follow‐up). The mutant allele fraction (MAF) was determined for each identified mutation using BEAMing.

**Results:**

A total of 68 of the 110 (61.8%) patients experienced a PFS event. Twenty‐six patients (23.6%) presented with an emergent p.T790M mutation in plasma at some point during follow‐up, preceding radiologic progression with a median of 76 (interquartile ratio: 54–111) days. Disease progression correlated with the appearance of p.T790M in plasma with a hazard ratio (HR) of 1.94 (95% confidence interval [CI], 1.48–2.54; *p* < 0.001). The HR for progression in patients showing increasing plasma sensitizing mutation levels (positive MAF slope) versus patients showing either decreasing or unchanged plasma mutation levels (negative or null MAF slopes) was 3.85 (95% CI, 2.01–7.36; *p* < 0.001).

**Conclusion:**

Detection and quantification of *EGFR* mutations in circulating tumor DNA using the highly sensitive BEAMing method should greatly assist in optimizing treatment decisions for advanced NSCLC patients.

## Graphical abstract

The manuscript is a multicenter, prospective study conducted in the real‐world setting. The study reveals that the detection and quantification (MAF slope) of EGFR mutations in ctDNA using the highly sensitive BEAMing method may assist in optimizing treatment decisions for advanced NSCLC patients.

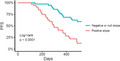

## 1 INTRODUCTION

Epidermal growth factor receptor (*EGFR*) genotyping is a routine test to assess non‐small‐cell lung cancer (NSCLC) patients’ eligibility to receive EGFR tyrosine kinase inhibitor (TKI) therapy. An additional *EGFR* mutation, known as exon 20 p.T790 M (p.T790 M), has been shown to be the most common cause of resistance to first‐ and second‐generation EGFR‐TKIs; it has been observed in 50%–60% of cases of acquired resistance.^1^ A meta‐analysis has shown that p.T790 M mutation emergence during EGFR‐TKI therapy has a negative impact on progression‐free survival (PFS),^2^ and since tumor tissue biopsy or re‐biopsy is not always viable or effective, analysis of circulating tumor‐derived DNA (ctDNA; also known as liquid biopsy) is a feasible alternative to tissue‐based *EGFR* mutation testing. Plasma‐based *EGFR* mutation detection is, therefore, a minimally invasive method for therapy response monitoring in NSCLC patients.^3^, ^4^ Finally, *EGFR* mutation results detected via liquid biopsy can precede radiologic progression by weeks, and in some cases, even months.^5^

As of the present day, few prospective trials have comprehensively monitored both sensitizing and resistance mutations in plasma samples during first‐ and second‐generation TKI treatment.^6^, ^7^, ^8^, ^9^ With respect to *EGFR* p.T790 M, for instance, it has been demonstrated that the risk of disease progression during therapy with first‐ or second‐generation EGFR‐TKIs is known to increase when the mutant allele fraction (MAF) of a preexisting p.T790 M mutation attains a threshold of ≥3.2%.^10^ Therefore, ascertaining MAF from the analyses of plasma‐detected *EGFR* sensitizing mutations is likely to improve gauging the prognosis of patients receiving EGFR‐TKIs. For example, a reduced benefit of EGFR‐TKI therapy has been associated with increasing levels of sensitizing mutations in ctDNA, likely reflecting an increase in systemic tumor burden and/or overall tumor activity.^9^ These findings underscore the importance of detecting and quantifying *EGFR* mutations in NSCLC patients before and during TKI therapy with the aim of optimizing the therapeutic regimen.^11^

Although third‐generation TKIs such as osimertinib are approved for use in the first‐line setting, their adoption into routine clinical practice varies widely.^12^ Furthermore, first‐ and second‐generation EGFR‐TKIs are prominently used in several cancer care settings—therefore, it remains a matter of urgency to be able to accurately and sensitively recognize the presence of p.T790 M in NSCLC patients treated with these targeted therapies, at the earliest possible time point. By detecting p.T790 M mutation as the most likely harbinger of treatment resistance that drives disease progression, an informed decision can be made for the patient to switch to osimertinib or an alternative therapy immediately upon the radiologic confirmation of disease progression. A highly sensitive liquid biopsy platform ideally suited for p.T790 M resistance detection is the digital polymerase chain reaction (PCR)‐based assay BEAMing (beads, emulsions, amplification, and magnetics). This assay reliably achieves a low limit of detection (0.02% MAF)^13^ and has been widely utilized in clinical settings.^14^, ^15^, ^16^, ^17^, ^18^, ^19^

The main goal of the LungBEAM study was to provide a pragmatic and real‐world approach to evaluate the value of EGFR mutation testing, using BEAMing technology, to monitor resistance and sensitizing mutations in plasma ctDNA and predict disease progression. An underlying imperative was to examine the correlation between the levels of *EGFR* sensitizing and p.T790 M mutations in plasma during the course of therapy and patient disease trajectory and survival after EGFR‐TKI treatment.

## 2 MATERIALS AND METHODS

### 2.1 Study design and patients

LungBEAM was a multicenter, prospective study that used BEAMing technology for mutation analysis in plasma ctDNA in stage IV NSCLC patients. Recruitment was conducted in 19 Spanish centers from November 2015 to May 2017. Eligible patients included adults with stage IV NSCLC, with a locally confirmed EGFR‐TKI sensitizing mutation (ex19del and/or L858R) by tissue biopsy, eligible to receive EGFR‐TKI therapy, and without prior treatment with EGFR‐TKIs or chemotherapy. Patients having concomitant malignancies were excluded. The study was approved by the institutional review board of each site and was conducted according to the principles of the Declaration of Helsinki. All eligible patients signed the informed consent form before any study‐specific procedures, sampling, or analyses.

### 2.2 Study procedures

Plasma samples were obtained from 10 ml of blood collected prior to any therapeutic intervention and every 4 weeks during treatment until disease progression, death, or completion of the study follow‐up (72 weeks after treatment initiation). After collection, plasma samples were frozen and then sent to either the Sysmex Inostics GmbH laboratory in Hamburg (Germany) or the Sysmex Inostics Inc CLIA laboratory in Baltimore (MD, USA) for *EGFR* mutation testing using the OncoBEAM^®^ EGFR assay. The OncoBEAM^®^ EGFR assay, based on BEAMing technology, detects eight *EGFR* mutations (p.T790 M, L858R, and six common del19 variants). The MAF was recorded. Tissue‐based mutation profiles were determined using the standard‐of‐care reference methodology used routinely in each hospital laboratory. For any discordant cases, BEAMing was also performed on tissue samples, when possible, to provide adjudication of results.

Disease progression was assessed according to the Response Evaluation Criteria in Solid Tumors (RECIST) 1.1 criteria. Computed tomography (CT) scans were performed every 8 weeks within the first 6 months and subsequently every 12 weeks (Table S1), unless any clinical condition required more frequent imaging. Investigators were blinded to plasma BEAMing results. All treatments and diagnostic procedures were performed according to the standard clinical practice in each center.

### 2.3 Statistical analysis

A total of 100 patients (72 PFS events expected at 18‐month follow‐up) were estimated to be included in the study to achieve 80% statistical power to detect the effect of the p.T790 M mutation at the 95% confidence level. We assumed that (i) the maximum time to progression is about 16 months in patients with an *EGFR* sensitizing mutation who receive first‐ or second‐generation EGFR‐TKI therapy^1^; (ii) about 50% of patients will develop a p.T790 M mutation^1^, ^20^; and (iii) the hazard ratio (HR) for PFS associated with the incidence of a p.T790 M mutation is around 2.^2^

Categorical variables are presented using absolute and relative frequencies, and continuous variables by dispersion measures, mainly median and interquartile range (IQR). PFS was defined as the time elapsed from the date of treatment initiation to disease progression or death, or, alternatively, to the date of the last blood sample collected within the 72‐week follow‐up period, whichever came first. A PFS event was defined as disease progression or death. Comparisons of clinical variables in patients with or without p.T790 M mutation were performed using the Chi‐square (*χ* ^2^) test, *t*‐test, or Mann–Whitney *U* test. Univariate and multivariate Cox models adjusted to clinical risk factors were used to investigate the effect of *EGFR* mutations detected in plasma on the PFS. In all models, the proportional hazard assumption was tested using Schoenfeld residuals.

Agreement between plasma and tissue *EGFR* mutation results was determined by Cohen's Kappa statistic. The slope of post‐baseline MAF of *EGFR* sensitizing mutations, defined as the mean change in MAF value for each patient, was calculated to characterize patient subgroups according to the differential patterns of progression in plasma mutation levels (measured in MAF), which were compared using the Wilcoxon Rank Sum test. Positive and negative slopes indicate increasing and decreasing MAF values, respectively, over time. Time‐to‐progression according to MAF patterns was analyzed using the Kaplan–Meier method and log‐rank testing.

All statistical tests were performed with two‐sided 95% confidence interval (CI) and 5% significance level. Statistical analyses were performed using the R 3.5.2 statistical software.

## 3 RESULTS

### 3.1 Study population

A total of 110 patients with stage IV *EGFR* mutated NSCLC met the selection criteria and were included in the study. The baseline characteristics of the cohort are shown in Table 1. The majority of patients were women (71.8%), non‐smokers (61.8%), and presented with synchronous diagnosis of primary tumor and metastatic disease (88.2%). A total of 22.7% of patients had brain metastasis at baseline. Tissue *EGFR* mutation distribution was 64.5% ex19del and 35.5% L858R; one patient (0.9%) harbored a tissue p.T790 M mutation at baseline.

**TABLE 1:** Summary of baseline patient and tumor characteristics and mutational analyses

Variable, n (%)	All patients (N=110)
Age, years^a^	65.5 (12.5)
Gender	
Male	31 (28.2)
Female	79 (71.9)
Smoking status	
Smoker	9 (8.2)
Ex‐smoker (1–5 years ago)	11 (10.0)
Ex‐smoker (6–10 years ago)	3 (2.7)
Ex‐smoker (>10 years ago)	19 (17.3)
Never	68 (61.8)
ECOG	
0	50 (45.5)
1	50 (45.5)
2	8 (7.3)
Not available	2 (1.8)
Stage M (at primary tumor diagnosis)	
M0	13 (11.8)
M1	97 (88.2)
Metastasis location	
M1a	28 (25.5)
M1b	78 (74.5)
Number of metastatic locations	
1	56 (50.9)
≥2	54 (49.1)
Tissue biopsy	
Primary tumor	85 (77.3)
Metastasis	25 (22.7)
*EGFR* mutation (tissue)	
Ex19del	71 (64.5)
L858R	39 (35.5)
p.T790 M mutation (tissue)	
Present	1 (0.9)
Absent	51 (46.4)
Not evaluated	58 (52.7)
Progression type	
Extrapulmonary	22 (38.6)
Pulmonary	17 (29.8)
Both	14 (24.6)
Not available	4 (7.0)

Abbreviations: ECOG, Eastern Cooperative Oncology Group; SD, standard deviation.

^a^ Data are presented as mean (SD).

**FIGURE 1:**
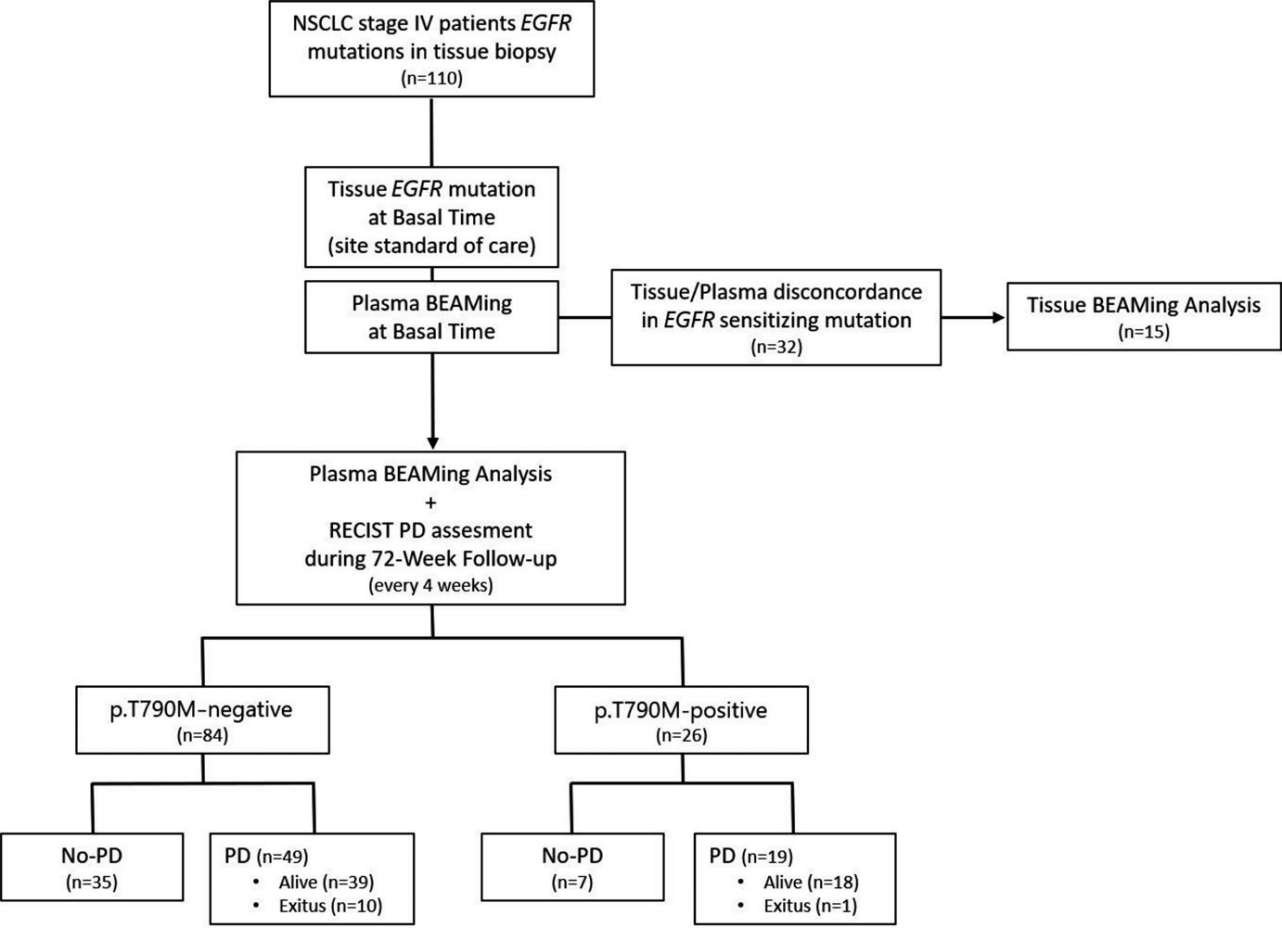
Study flow‐chart. *EGFR*, epidermal growth factor receptor; NSCLC, non‐small cells lung cancer; PD, progressive disease; RECIST, Response Evaluation Criteria In Solid Tumors

During the 72‐week follow‐up period, a total of 68 (61.8%) patients experienced a PFS event (Table 1; Figure 1). Follow‐up was lost or incomplete in seven cases. Overall, the median (IQR) follow‐up was 352 (164–504) days.

### 3.2 Concordance between plasma BEAMing results with those from tissue testing

Baseline *EGFR* sensitizing mutations were detected in the plasma of 70.9% (78/110) of the patients. Of these baseline plasma sensitizing mutation‐positive patients, 67.9% (53/78) presented with ex19del, while 32.1% (25/78) had L858R mutations. The overall sensitivity for detection of plasma sensitizing mutations by BEAMing was 70.9% (95% CI, 61.4–79.0). In patients with baseline sensitizing mutations detected in plasma, the concordance of plasma results with those obtained from tissue testing was 98.7% (Kappa: 97%; 95% CI, 91.2–102.8; Table S2).

Among the 32 patients showing different tissue and plasma *EGFR* mutation results (positive tissue and negative plasma), additional BEAMing tests were performed on 15 available initial specimens used for standard‐of‐care tissue testing, and valid data were obtained in 14 cases. The concordance of the *EGFR* mutations detected by the BEAMing technique in tissue with the standard‐of‐care tissue result was 100% (95% CI, 71.1–100; Table S2).

The presence or absence of a p.T790 M resistance mutation in tissue at baseline was only able to be assessed in 52 patients—this was due to the limitations of the reference method in some centers. The sensitivity of BEAMing to detect p.T790 M in plasma was 100% (95% CI, 100–100; Table S2). The concordance between tissue and plasma for the detection of p.T790 M at baseline was 100%, with a Kappa index of 100% (95% CI, 100–100).

### 3.3 Detection of p.T790 M mutation in plasma and risk of disease progression

Twenty‐six patients (23.6%) had a p.T790 M mutation detected in their plasma at some point during follow‐up. Of these, 19 (73.1%) had a PFS event during the study period. Of all enrolled patients who progressed (68 patients), 19 (27.9%) presented with a p.T790 M mutation in their plasma during the 72‐week follow‐up. There were no significant differences in demographic or clinical‐pathologic factors between plasma p.T790 M‐positive and p.T790 M‐negative patients (Table S3). The median of maximum MAF values in p.T790 M‐positive patients was 0.62% (IQR: 0.15–1.88). Significant differences in plasma *EGFR* sensitizing mutation MAF levels were observed between patients with or without p.T790 M (0.20 vs. 2.50; *p* < 0.001), regardless of the initial baseline level of sensitizing mutations (Table S4).

**FIGURE 2:**
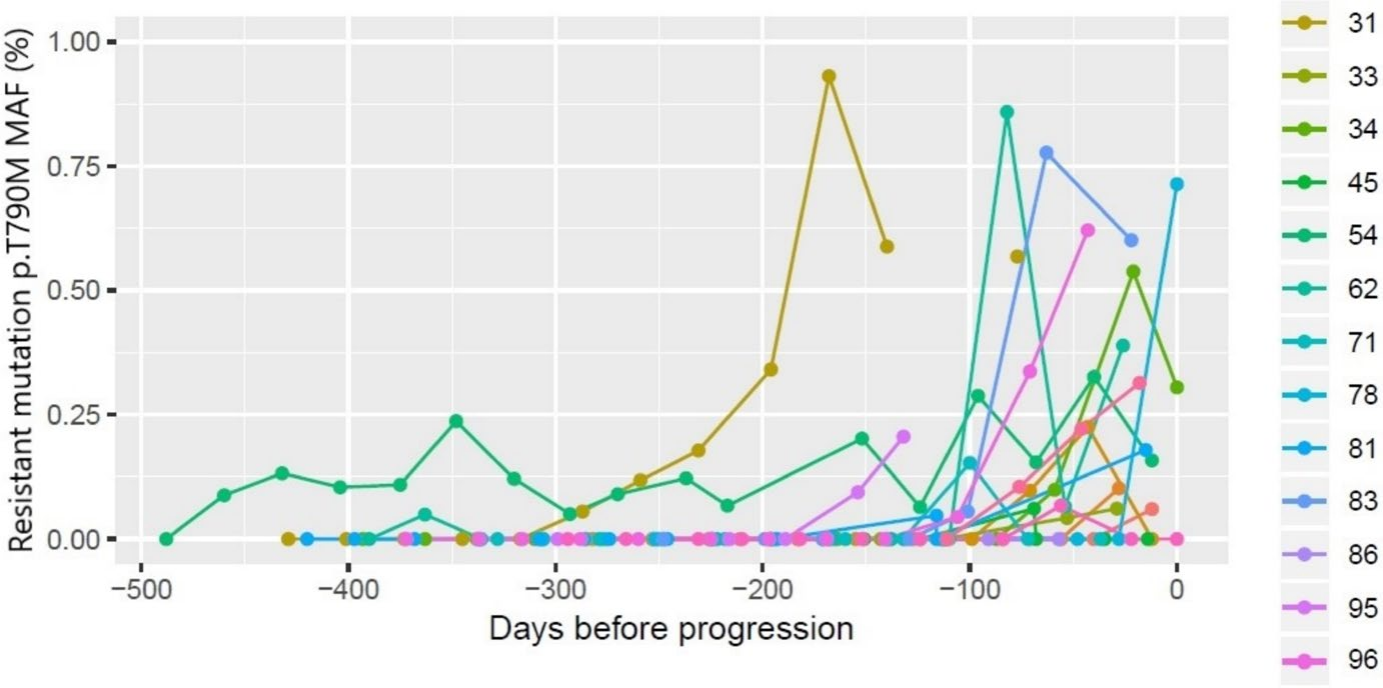
MAF values profile in p.T790 M‐positive patients with progression disease. The event day was taken as a reference to better clarify the evolution of MAF values toward the event time point. One patient was removed to scale the graph because of its high values (up to 12%). MAF, mutant allele fraction

The finding of a p.T790 M resistance mutation in plasma preceded the corresponding PFS event by a median of 76 (IQR: 54–111) days. In seven (36.8%) of these patients, the p.T790 M mutation was detected within the first 16 weeks of follow‐up (Table S5). Of note, one patient (ID_10) presented with, and persistently showed, high p.T790 M levels (42.9% MAF at baseline); this patient was confirmed to harbor a germline p.T790 M mutation and did not experience disease progression at any point during the 72‐week follow‐up (Figure S1; Table S5). Plasma monitoring revealed an overall increase in p.T790 M MAF values up until radiologic progression (Figure 2; Table S6).

The HR for disease progression associated with detection of p.T790 M resistance mutations in plasma was 1.94 (95% CI, 1.48–2.54; *p* < 0.001). This effect remained significant even after the addition of other risk factors as confounders (e.g., gender, smoking or multiple metastases, HR: 2.08; 95% CI, 1.58–2.74; Table S7). After incorporation of plasma sensitizing mutations as predictors, the effect of p.T790 M was decreased but remained significant (HR: 1.38; 95% CI, 1.02–1.87; *p* < 0.038; Table 2). Detection of sensitizing mutations in plasma by BEAMing was significantly associated with disease progression when adjusted for other risk factors (Table 2). There was a higher impact of the mutation on disease progression with mutation Ex19del in exon 19, where the HR for disease progression was 2.12 (95% CI 1.67–2.70), than with mutation L858R in exon 21 (HR 1.68; 95% CI 1.27–2.22, both *p* < 0.001).

**TABLE 2:** Risk factors of progression during the 72‐week follow‐up in multivariate Cox regression analysis

Variable	HR (95% CI)	*p* value
Age	1.02 (1.01–1.03)	<0.001
Man	1.06 (0.87–1.29)	0.584
Smoker	1.42 (1.03–1.94)	0.030
Ex‐smoker (1–5 years ago)	1.53 (1.13–2.06)	0.006
Ex‐smoker (6–10 years ago)	0.77 (0.43–1.38)	0.379
Ex‐smoker (>10 years ago)	1.00 (0.78–1.28)	0.992
Metastasis (>1 location)	1.27 (1.06–1.53)	0.011
p.T790 M Mutation	1.38 (1.02–1.87)	0.038
Ex19del	2.12 (1.67–2.70)	<0.001
L858R	1.68 (1.27–2.22)	<0.001

Abbreviations: CI, confidence interval; HR, hazard ratio.

### 3.4 Detection of sensitizing mutations in plasma and risk of disease progression

As stated above, baseline plasma sensitizing mutations were detected in 70.9% (78/110) of patients enrolled in the study (Figure S2). The median baseline MAF detected in these patients was 0.79% (IQR: 0.22–7.56). Among these patients, 53 (67.9%) had a PFS event during the 72‐week follow‐up period (Table S8). No significant differences in the distribution of sensitizing mutations with respect to both mutation type and exon location were observed between patients with or without PFS events.

**FIGURE 3:**
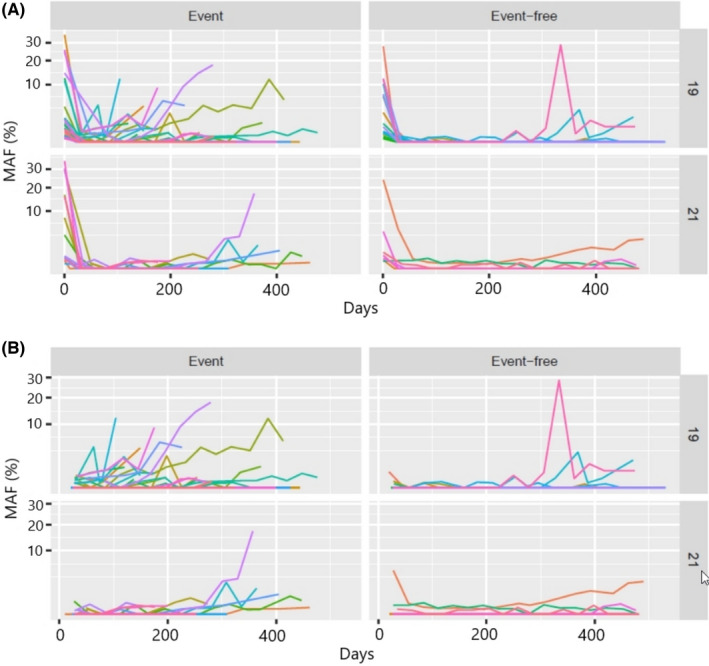
Progression of MAF values stratified by sensitizing mutation exon type and patient outcome (PFS event). A square root scale of MAF values was used to better visualize MAF progression in patients with very low values. A, Baseline and 4 weekly follow‐up MAF values. B, Baseline values are excluded to clarify MAF patterns. MAF, mutant allele fraction; PFS, progression‐free survival

Among the 78 patients with plasma baseline *EGFR* sensitizing mutations, 68 of them had at least four post‐baseline samples available and were used to assess the longitudinal patterns of *EGFR* mutation MAF levels. A marked reduction of MAF values from baseline levels was observed early during treatment in all patients (Figure 3A), dropping to zero in some cases. After this initial precipitous drop in MAF, most patients with a PFS event showed a rebound, with MAF values increasing progressively at a variable rate. In contrast, most PFS event‐free patients showed relatively few changes in MAF levels, although some random variations were observed (Figure 3B). Patients with PFS events tended to have significantly higher MAF slope values than event‐free patients (Wilcoxon Rank Sum Test *p* < 0.001). Most patients with PFS events (65.1%; 28/43) showed positive MAF slope values, whereas most event‐free patients had negative or null slopes (84.0%; 21/25).

**FIGURE 4:**
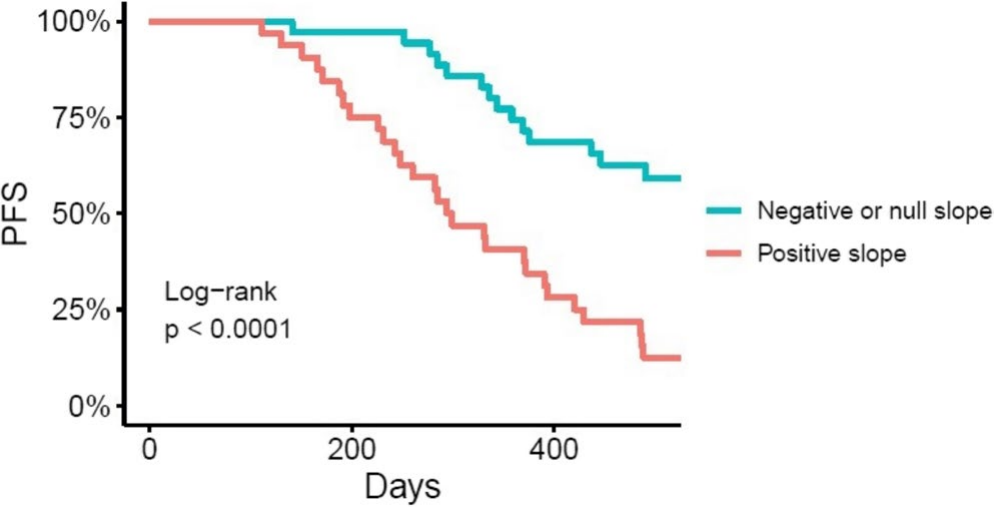
PFS according to the slope (mean increase) of MAF in patients with a confirmed *EGFR* sensitizing mutation in plasma at baseline. *EGFR*, epidermal growth factor receptor; MAF, mutant allele fraction; PFS, progression‐free survival

Progression‐free survival was shorter in patients showing a positive MAF slope than in those with a negative or null slope (Figure 4). The median PFS was 296.5 (IQR: 248–393) days in patients with a positive slope, but non‐estimable in patients with a negative or null slope. The Kaplan–Meier estimates of 1‐year PFS rates were 40.6% (95% CI, 26.7–61.8) and 74.3% (95% CI, 61.2–90.3) for patients with positive and negative/null slopes, respectively.

The HR for disease progression in patients with a positive MAF slope was 3.85 (95% CI, 2.01–7.36; *p* < 0.001). The effect remained significant when adding exon type as a confounder (HR: 4.11; 95% CI, 2.11–8.03; *p* < 0.001). No effect of exon type on PFS was observed (HR: 1.10; 95% CI, 0.57–2.13; *p* = 0.765). In the multivariate model, there was no association between mutated *EGFR* exon type and PFS (HR: 0.76; 95% CI, 0.39–1.49; *p* = 0.423).

## 4 DISCUSSION

Precision medicine in the setting of NSCLC is now routine due to molecular profiling strategies, which can identify gene alterations in a patient's tumor that are targeted by effective therapies. Due to the drawbacks of tissue biopsy (and re‐biopsy) for identifying and monitoring *EGFR* mutations, the liquid biopsy approach is a viable, non‐invasive, rapid, and accurate alternative for detecting both *EGFR* sensitizing and resistance mutations. Plasma *EGFR* mutation testing with rapid turnaround time enables physicians to quickly identify those patients with *EGFR* mutation‐positive disease, enabling early and optimal treatment of these patients that ultimately improves outcomes.^21^ In this real‐world LungBEAM study, almost 62% of stage IV NSCLC patients with *EGFR* sensitizing mutations that received first‐ and second‐generation TKI treatment as first‐line therapy had a PFS event during the 72‐week follow‐up period, with 27.9% of patients showing an emergent plasma p.T790 M mutation detected using BEAMing at some points during the follow‐up. This percentage of patients was lower than reported in previous studies; however, this could be attributed to the considerably shorter duration of follow‐up in our study (72 weeks from the initiation of EGFR‐TKI therapy) compared with these other studies.^1^, ^22^, ^23^

The emergence of the *EGFR* p.T790 M mutation during EGFR‐TKI treatment was associated with a shorter PFS, and this was independent of other clinical factors. This result is in contrast to those of previous reports indicating that persistence of p.T790 M expression represents indolent progression.^20^ Notably, our results showed that p.T790 M detection in plasma occurs at a median of 76 days prior to radiological disease progression. In addition, there was a distinct increase in p.T790 M MAF values prior to or at the time of disease progression in p.T790 M‐positive patients. In one‐third of these patients, the p.T790 M mutation was detected during the first 16 weeks of TKI treatment. Early detection of p.T790 M mutation in plasma is important because it helps identify patients that may benefit from earlier and more frequent imaging to monitor disease progression, which can better inform subsequent therapeutic interventions without the need for an invasive tissue biopsy procedure.^24^, ^25^ In addition, the evidence shown in the present study indicates that the trajectory of increasing *EGFR* mutation levels (MAF) is an important signal that a patient's tumor burden is increasing.^26^, ^27^ This principle has also been observed during the monitoring of colorectal cancer patients.^28^, ^29^ Additional studies will be required to fully explore the prognostic impact of increasing plasma *EGFR* mutation levels in NSCLC.

At the initiation of this study, third‐generation EGFR‐TKIs such as osimertinib were under experimental evaluation as a potential second‐line treatment after first‐ and second‐generation EGFR‐TKIs such as erlotinib. Hence, the initial objective was to monitor the early appearance of *EGFR* p.T790 M mutation in plasma, a well‐established mechanism of resistance to first‐ and second‐generation TKIs. Although the clinical application of plasma p.T790 M detection and monitoring has fallen as a result of the increase in the use of third‐generation TKIs as first‐line treatment,^30^ it is nonetheless still vital to detect p.T790 M mutations in the various treatment settings where first‐ and second‐generation TKIs remain in use.^31^ Moreover, sequential use of second‐generation EGFR‐TKIs followed by osimertinib might be one of the most practical ways of improving overall survival in patients that develop p.T790 M resistance. There are a number of current clinical trials exploring sequential EGFR‐TKI treatments, as well as investigating novel therapeutic strategies for patients with resistance due to p.T790 M,^30^, ^31^ and the paradigm of sensitive p.T790 M detection demonstrated in LungBEAM is expected to be highly useful in these settings.

Regarding the detection of resistance to first‐line osimertinib treatment, it should be noted that BEAMing technology can also be used to detect the *EGFR* C797S mutation, which is a frequent mechanism of resistance after first‐line administration of osimertinib.^32^ However, other resistance mechanisms including variations in *C*‐*MET* copy number variation, as well as cell cycle gene amplifications, *BRAF* mutations, *PI3KCA* mutations, and human epidermal growth factor receptor‐2 amplification/mutations,^33^, ^34^ necessitate a broader coverage of these gene alterations. Hence, next‐generation sequencing‐based methods may prove to be more useful in identifying these cases of therapy resistance, as they allow the simultaneous detection of more of these alterations in multiple genes.^35^ However, the levels of clinical sensitivity for the detection of these alterations vary considerably and are challenging to report with accuracy. Therefore, considerable care should be exercised in interpreting such data used to support treatment recommendations.^36^

Regarding the detection of *EGFR* sensitizing mutations, the baseline and MAF levels of these mutations in plasma samples obtained by BEAMing prior to the initiation of EGFR‐TKI therapy were significantly associated with a lower PFS, both for ex19del and L858R, even after adjusting for other risk factors. These findings suggest that plasma *EGFR* mutation testing in combination with a tissue test at baseline can provide important complementary information regarding patient prognosis. Moreover, the tracking of sensitizing mutation levels in plasma over time showed distinctly different patterns depending on whether or not the patient showed disease progression, indicating that the changing levels of MAF over time are useful in the monitoring of treatment response.^37^, ^38^ Patients with a persistent increase in MAF values after the post‐baseline drop were prone to progress during the follow‐up. The patient‐specific post‐baseline MAF slope (mean change) is a measure of the rate of MAF increase observed after the initial drop. These slopes allowed us to classify patients into two groups: those who had a progressive increase in MAF values (positive slope) and those who did not (null or negative slopes). Comparison of these two groups of patients showed that they experienced clinically meaningful differences in 1‐year PFS rates: these rates were significantly lower in those with a positive slope versus those with a null or negative slope (40.6% vs. 74.2%). Thus, monitoring plasma *EGFR* mutations and MAF levels using BEAMing during the follow‐up period enabled accurate measurement of a patient's response to treatment. In line with this idea, it has been proposed that sensitizing mutations, such as ex19del, can accurately reflect tumor biology, and it could be used as a sensitive biomarker to monitor disease outcomes.^14^, ^15^

Another important consideration in interpreting our study results is that mutations detected by the analysis of ctDNA should be regarded as time‐dependent variables.^39^ In fact, our MAF progression analysis was restricted to the group of patients with at least four post‐baseline plasma samples available, so those patients with early progression were not included. Increasing the frequency of plasma monitoring (e.g., weekly or every 2 weeks) might show even greater utility of plasma BEAMing *EGFR* mutation monitoring, especially in patients that are suspected to be at high risk for progression. In light of this, the *EGFR* mutation profile in NSCLC patients should be determined quickly, given that the early detection of circulating *EGFR* mutations in plasma, as well as increasing mutation levels, is prognostic of rapid tumor progression, and should signal the opportunity to consider transitioning to a different treatment.

Although the value of liquid biopsy to ascertain tumor mutation status is well established,^35^, ^40^, ^41^ there are some barriers to its widespread use, such as concerns about false‐negative results. In lung cancer, false‐negative results can occur more frequently in plasma testing as compared to tissue testing, because the shedding of ctDNA into the blood in NSCLC patients has been observed to be lower than in patients with other solid tumors. In colorectal cancer, for instance, the concordance of mutation results derived from plasma and tissue is equal to or greater than 90%.^42^ Nevertheless, the use of the BEAMing technology in LungBEAM, as well as in other studies,^13^, ^19^, ^43^ has shown high sensitivity in detecting *EGFR* mutations in plasma, which makes it a feasible alternative to tissue‐based mutation analysis in order to guide therapy selection and response evaluation for NSCLC patients.

## 5 CONCLUSION

BEAMing accurately detected the presence of *EGFR* mutations and showed that increasing MAF values for sensitizing and p.T790 M mutations in serial plasma samples are an important negative prognostic indicator. Future studies should be pursued to determine a relevant clinical cut‐off point for MAF for use as an early disease progression prognostic tool, independent of imaging results, in patients with metastatic NSCLC.

## 6 ETHICAL APPROVAL STATEMENT

The study was approved by the institutional review board at each hospital and was conducted in accordance with the principles of the Declaration of Helsinki. All eligible patients signed the informed consent form prior to any study‐specific procedures, sampling, or analyses.

## CONFLICT OF INTEREST

JMST, AP, MLB, and JP declare no potential conflict of interests. The following authors declare the following financial interests/personal relationships which may be considered as potential competing interests: PG reports personal fees from Roche, MSD, BMS, Boehringer Ingelheim, Pfizer, Abbvie, Novartis, Lilly, AstraZeneca, Janssen Blueprint Medicines, Takeda, Gilead, Rovi, outside the submitted work; LPA has received honorarium from Adacap, Amgen, AstraZeneca, Bayer, Blueprint Medicines, Boehringer Ingelheim, Bristol‐Myers Squibb, Celgene, Eli Lilly, Incyte, Ipsen, Merck, Merck Sharp and Dohme, Novartis, Pfizer, PharmaMar, Roche, Sanofi, Servier, Sysmex, Takeda, and research grant/funding from AstraZeneca, Bristol‐Myers Squibb, Merck Sharp and Dohme, Pfizer; MM reports grants and personal fees from BMS, personal fees and non‐financial support from MSD, Boehringer Ingelheim, personal fees, non‐financial support and other from AstraZeneca, Roche, personal fees from Kyowa Kirin, Pierre Fabre, outside the submitted work; TM reports speaker honoraria from Roche, BMS, Boehringer Ingelheim, AstraZeneca, and has received a research grant from Kyowa Kirin; JMT reports fees for consulting/advisory role from MSD, BMS, Bayer, EISAI, AstraZeneca, and speaker honoraria from Bayer, BMS, Takeda; JBB reports grants and personal fees from Roche‐Genentech, grants from Pfizer and Pierre Fabre, and personal fees from MSD, BMS, AstraZeneca, and Novartis, outside the submitted work; RGC reports consulting/advisory role honoraria from AstraZeneca, Novartis, Pfizer, Boehringer Ingelheim, Roche/Genentech, BMS, Takeda, MSD Oncology, Janssen Oncology, and speaker honoraria from MSD Oncology, MSD, Takeda, Roche; JLGL reports consulting/advisory role honoraria from Janssen‐Cilag, MSD, BMS, Boehringer Ingelheim, speaker honoraria from MSD, and research funding from Mirati Therapeutics, AstraZeneca, Bayer, OncoMed, Astellas Pharma, Janssen‐Cilag, Roche, Abbvie, Boehringer Ingelheim, Pfizer, PharmaMar, BMS, Novartis, Celgene, Ignyta; DI reports consulting honoraria from AbbVie, Amgen, AstraZeneca, BMS, Boehringer Ingelheim, Eli Lilly Oncology, F. Hoffmann‐La Roche, Merck, MSD, Novartis, Pierre Fabre, Pfizer, Takeda, speaker honoraria from Amgen, AstraZeneca, BMS, Boehringer Ingelheim, Eli Lilly Oncology, F. Hoffmann‐La Roche, MSD, Novartis, Pierre Fabre, Pfizer, and research grants from AstraZeneca, BMS, F. Hoffmann‐La Roche, MSD, Pierre Fabre; NV reports grants from Roche, during the conduct of the study, personal fees from Roche, other from BMS, personal fees from Pfizer, other from Boehringer Ingelheim, personal fees and other from Lilly, other from AstraZeneca, outside the submitted work; CC has received speaker honoraria from AstraZeneca, Roche, MSD, Pfizer, Bristol‐Myers Squibb, Takeda, for advisory role from AstraZeneca, Bristol‐Myers Squibb, MSD, Roche, Bayer, Angelini Research, and funding from AstraZeneca, Bristol‐Myers Squibb; AI has received honoraria for consulting or advisory role from BMS, Boehringer Ingelheim, MSD, Pfizer, Roche, and for expert testimony from AstraZeneca, Boehringer Ingelheim, MSD, Pfizer, Roche; OJ has received honoraria for consulting or advisory role from Boehringer Ingelheim, Bristol‐Myers Squibb, Merck Sharp & Dohme, Roche/Genentech, Abbvie, and research funding from Bristol‐Myers Squibb, AstraZeneca; BM reports grants and personal fees from Roche, personal fees and other from BMS, Takeda, other fees from MSD, Boehringer Ingelheim, outside the submitted work; AA reports speaker honoraria from AstraZeneca and for consulting/advisory role from Boehringer Ingelheim; EF reports personal fees from Abbvie, AstraZeneca, Blue Print Medicines, Boehringer Ingelheim, BMS, Eli Lilly, Guardant Health, Janssen, Medscape, Merck KGaA, MSD, Novartis, Pfizer, Prime Oncology, Roche, Samsung, Takeda, Touchime, GSK, Bayer, has received grants from Grant for Oncology Innovation (GOI) and Fundación Merck Salud, and she is an Independent Member of the Board in Grifols, outside the submitted work.

## AUTHOR CONTRIBUTION

PG: Conceptualization, Methodology, Investigation, Supervision, Writing ‐ Original Draft, Writing‐ Reviewing and Editing. LPA: Conceptualization, Methodology, Investigation, Supervision, Writing ‐ Original Draft, Writing‐ Reviewing and Editing. MM: Investigation, Writing ‐ Reviewing and Editing. TM: Investigation, Writing ‐ Reviewing and Editing. JMT: Investigation, Writing ‐ Reviewing and Editing. JBB: Investigation, Writing ‐ Reviewing and Editing. RGC: Investigation, Writing ‐ Reviewing and Editing. JLGL: Investigation, Writing ‐ Reviewing and Editing. JMST: Investigation, Writing ‐ Reviewing and Editing. DI: Investigation, Writing ‐ Reviewing and Editing. NV: Investigation, Writing ‐ Reviewing and Editing. CC: Investigation, Writing ‐ Reviewing and Editing. AI: Investigation, Writing ‐ Reviewing and Editing. OJ: Investigation, Writing ‐ Reviewing and Editing. BM: Investigation, Writing ‐ Reviewing and Editing. AP: Investigation, Writing ‐ Reviewing and Editing. AA: Investigation, Writing ‐ Reviewing and Editing. MLB: Investigation, Writing ‐ Reviewing and Editing. JP: Conceptualization, Methodology, Investigation, Supervision, Writing ‐ Original Draft, Writing ‐ Reviewing and Editing. EF: Conceptualization, Methodology, Investigation, Supervision, Writing ‐ Original Draft, Writing ‐ Reviewing and Editing.

## Supporting information

Supplementary Material

## Data Availability

The data that support the findings of this study are available on request from the corresponding author. The data are not publicly available due to privacy or ethical restrictions.
