# Supplementary material for: LungBEAM: A prospective multicenter study to monitor stage IV NSCLC patients with EGFR mutations using BEAMing technology
Source: Cancer Med. 2021 Jul 23;10(17):5878–88. doi: 10.1002/cam4.4135 (PMC8419773; doi:10.1002/cam4.4135)
Supplement: Supplementary file 1 — Supplementary Material [file CAM4-10-5878-s001.docx]

**LungBEAM: A prospective multicenter study to monitor stage IV NSCLC patients with *EGFR* mutations using BEAMing technology**

**Supplementary materials**

***Contents***

**Table S1.** Study design and timetable of scheduled procedures.

**Table S2.** Performance of BEAMing analysis.

**Table S3.** Characteristics of patients according to p.T790M status in plasma.

**Table S4.** Median of maximum MAF values of plasma sensitizing mutation stratified by p.T790M co‑mutation.

**Table S5.** P.T790M-positive patients with early p.T790M detection in plasma.

**Table S6.** MAF values of p.T790M resistance mutation during follow-up.

**Table S7.** Multivariate analysis of p.T790M in plasma and other risk factors for progression.

**Table S8.** Type of *EGFR* sensitizing mutation detected in baseline plasma, stratified by patient outcome (PFS event).

**Figure S1.** Evolution of MAF in p.T790M-positive patients during the 72-week follow-up period, stratified by patient outcome (PFS event).

**Figure S2.** Description and ratio of sensitizing mutation type detected in plasma samples during the 72-week follow-up.

**Table S1** Study design and timetable of scheduled procedures.

|  | Study Period | | | | | | | | | | | | | | | | | | |
| --- | --- | --- | --- | --- | --- | --- | --- | --- | --- | --- | --- | --- | --- | --- | --- | --- | --- | --- | --- |
| Time (weeks) | **0** | **4** | **8** | **12** | **16** | **20** | **24** | **28** | **32** | **36** | **40** | **44** | **48** | **52** | **56** | **60** | **64** | **68** | **72^a^** |
| Informed consent | ● |  |  |  |  |  |  |  |  |  |  |  |  |  |  |  |  |  |  |
| Selection criteria | ● |  |  |  |  |  |  |  |  |  |  |  |  |  |  |  |  |  |  |
| Tissue biopsy for *EGFR*/p.T790M mutations | ● |  |  |  |  |  |  |  |  |  |  |  |  |  |  |  |  |  |  |
| Liquid biopsy for *EGFR*/p.T790M mutations | ● | ● | ● | ● | ● | ● | ● | ● | ● | ● | ● | ● | ● | ● | ● | ● | ● | ● | ● |
| Clinical assessment | ● | ● | ● | ● | ● | ● | ● | ● | ● | ● | ● | ● | ● | ● | ● | ● | ● | ● | ● |
| CT scan |  |  | ● |  | ● |  | ● |  |  | ● |  |  | ● |  |  | ● |  |  | ● |

^a^End of study was at week 72, time of radiographic progression, or time of cessation of EGFR-TKI treatment.

Abbreviations: CT, computed tomography; *EGFR*, epidermal growth factor receptor.

**Table S2** Performance of BEAMing analysis. (A) Concordance between plasma BEAMing and tissue analysis by standard of care for mutation type detected at baseline. (B) Concordance between tissue BEAMing and standard of care for sensitizing mutations type detected at baseline performed on discordant cases.

| **(A)** |  | |  |  |  |  | |  |
| --- | --- | --- | --- | --- | --- | --- | --- | --- |
|  | **Tissue SM** | |  |  |  | **Tissue p.T790M** | | |
| **Plasma** | Ex19del | L858R |  |  | **Plasma** | MD | NMD |  |
| Ex19del | 53 | 0 | **53** |  | MD | 1 | 0 | 1 |
| L858R | 1 | 24 | **25** |  | NMD | 0 | 51 | 51 |
|  | **54** | **24** | **78** |  |  | **1** | **51** | **52** |
|  |  |  |  |  |  |  |  |  |
| **(B)** |  | |  |  |  |  | |  |
|  | **Tissue SM** | |  |  |  |  | |  |
| **Tissue**  **BEAMing** | Ex19del | L858R |  |  |  |  |  |  |
| Ex19del | 6 | 0 | **6** |  |  |  |  |  |
| L858R | 0 | 8 | **8** |  |  |  |  |  |
|  | **6** | **8** | **14** |  |  |  |  |  |

Abbreviations: MD, mutation detected; NMD, no mutation detected; SM sensitizing mutations.

**Table S3** Characteristics of patients according to p.T790M status in plasma.

|  | **NMD**  **(n = 84)** | **MD**  **(n = 26)** | ***P* value** |
| --- | --- | --- | --- |
| **Gender** |  |  | 0.391 |
| Man | 26 (31.0%) | 5 (19.2%) |  |
| Woman | 58 (69.0%) | 21 (80.8%) |  |
| **Age, years^a^** | 66.1 (12.3) | 63.6 (13.1) | 0.404 |
| **Smoking habit** |  |  | 0.391 |
| Smoker | 9 (10.7%) | 0 (0.00%) |  |
| Ex-smoker (1–5 years ago) | 8 (9.52%) | 3 (11.5%) |  |
| Ex-smoker (6–10 years ago) | 3 (3.57%) | 0 (0.00%) |  |
| Ex-smoker (> 10 years ago) | 15 (17.9%) | 4 (15.4%) |  |
| Never | 49 (58.3%) | 19 (73.1%) |  |
| **ECOG** |  |  | 0.611 |
| 0 | 40 (47.6%) | 10 (38.5%) |  |
| 1 | 37 (44.0%) | 13 (50.0%) |  |
| 2 | 5 (5.95%) | 3 (11.5%) |  |
| N/A | 2 (2.38%) | 0 (0.00%) |  |
| **Stage M at primary tumor diagnosis** |  |  | 0.729 |
| M0 | 11 (13.1%) | 2 (7.69%) |  |
| M1 | 73 (86.9%) | 24 (92.3%) |  |
| **Metastasis location** |  |  |  |
| Pulmonary | 44 (52.4%) | 13 (50.0%) | 1.000 |
| Bone | 36 (42.9%) | 15 (57.7%) | 0.271 |
| Hepatic | 12 (14.3%) | 5 (19.2%) | 0.544 |
| Brain | 21 (25.0%) | 4 (15.4%) | 0.450 |
| Adrenal | 8 (9.52%) | 2 (7.69%) | 1.000 |
| Subcutaneous | 1 (1.19%) | 0 (0.00%) | 1.000 |
| Other | 24 (28.6%) | 6 (23.1%) | 0.766 |
| **Number of metastatic locations** |  |  | 0.801 |
| Multiple | 41 (48.8%) | 13 (50.0%) |  |
| Single | 43 (51.2%) | 13 (50.0%) |  |
| Liver | 1 (2.3%) | 0 (0.0%) |  |
| Pulmonary | 19 (44.2%) | 5 (38.5%) |  |
| Cerebral | 9 (20.9%) | 1 (7.7%) |  |
| Bone | 10 (23.3%) | 6 (46.2%) |  |
| Other | 4 (9.3%) | 1 (7.7%) |  |
| **Tissue biopsy** |  |  | 0.450 |
| Primary tumor | 63 (75.0%) | 22 (84.6%) |  |
| Metastasis | 21 (25.0%) | 4 (15.4%) |  |
| **Basal tissue *EGFR* mutation** |  |  | 0.736 |
| Ex19del | 53 (63.1%) | 18 (69.2%) |  |
| L858R | 31 (36.9%) | 8 (30.8%) |  |
| **Basal tissue p.T790m mutation** |  |  | 0.324 |
| Present | 0 (0.00%) | 1 (3.85%) |  |
| Absent | 39 (46.4%) | 12 (46.2% |  |
| Not evaluated | 45 (53.6%) | 13 (50.0%) |  |

Data are presented as n (%)

^a^Mean (standard deviation)

Abbreviations: ECOG, Eastern Cooperative Oncology Group; *EGFR*, epidermal growth factor receptor; MD, mutation detected; NMD, no mutation detected.

**Table S4** Median of maximum MAF values of plasma sensitizing mutation stratified by p.T790M co‑mutation.

| **Sensitizing mutation in plasma** | **p.T790M-negative**  **(n = 84)** | **p.T790M-positive**  **(n = 26)** | ***P* value** |
| --- | --- | --- | --- |
| All | 0.20 (0.00–2.30) | 2.50 (1.10–11.5) | < 0.001 |
| Post-baseline^a^ | 0.00 (0.00–0.11) | 1.61 (0.26–7.23) | < 0.001 |

Data are presented as median (IQR).

^a^ The baseline data is excluded to capture elevations after the initial fall.

Abbreviations: IQR, interquartile range; MAF, mutant allele fraction.

**Table S5 p.**T790M-positive patients with early p.T790M detection in plasma.

| **Patient**  **ID** | **MAF (%) in early follow-up** | | | | | **Time prior to PD (weeks)** |
| --- | --- | --- | --- | --- | --- | --- |
|  | **Week 0**^a^ | **Week 4** | **Week 8** | **Week 12** | **Week 16** |  |
| 6 | NMD | – | NMD | NMD | 0.06 | 1.7 |
| 10^b^ | 42.937 | 47.808 | 46.136 | 44.919 | 47.036 | No PD |
| 45 | NMD | NMD | NMD | 0.061 | NMD | 9.9 |
| 50 | NMD | 0.059 | NMD | NMD | – | No PD |
| 54 | NMD | 0.088 | 0.132 | 0.104 | 0.109 | 65.7 |
| 59 | NMD | NMD | NMD | NMD | 0.06 | 11.3 |
| 62 | NMD | 0.049 | NMD | NMD | NMD | 51.9 |
| 86 | NMD | NMD | 1.104 | – | – | 4.0 |
| 113 | NMD | 0.105 | 0.222 | 0.314 | 0.704 | 10.9 |

^a^Week 0 = treatment initiation.

^b^Patient with germline p.T790M mutation.

Abbreviations: NMD, no mutation detected; MAF, mutant allele fraction; PD, progressive disease; (–) Plasma sample not available.

**Table S6** MAF values of p.T790M resistance mutation during follow-up.

| **Follow-up time (week)** | **p.T790M-positives with PD (n)^a^** | **Average (SD)** | **Median (IQR)** |
| --- | --- | --- | --- |
| 0 | 0 | 0.000 (0.000) | 0.000 (0.000-0.000) |
| 4 | 0 | 0.015 (0.034) | 0.000 (0.000-0.000) |
| 8 | 0 | 0.086 (0.269) | 0.000 (0.000-0.000) |
| 12 | 1 | 0.030 (0.081) | 0.000 (0.000-0.000) |
| 16 | 2 | 0.011 (0.030) | 0.000 (0.000-0.000) |
| 20 | 3 | 0.034 (0.068) | 0.000 (0.000-0.055) |
| 24 | 4 | 0.090 (0.208) | 0.000 (0.000-0.099) |
| 28 | 4 | 0.206 (0.593) | 0.000 (0.000-0.070) |
| 32 | 5 | 0.528 (1.470) | 0.067 (0.000-0.225) |
| 36 | 6 | 0.772 (2.166) | 0.044 (0.000-0.112) |
| 40 | 7 | 1.046 (2.966) | 0.000 (0.000-0.270) |
| 44 | 10 | 0.507 (0.488) | 0.621 (0.000-0.859) |
| 48 | 12 | 0.608 (0.731) | 0.385 (0.125-0.678) |
| 52 | 10 | 1.002 (1.357) | 0.464 (0.308-1.157) |
| 56 | 15 | 0.607 (0.701) | 0.296 (0.261-0.643) |
| 60 | 16 | 0.154 (na) | 0.154 (0.154-0.154) |
| 64 | 17 | 0.326 (na) | 0.326 (0.326-0.326) |
| 68 | 18 | 0.158 (na) | 0.158 (0.158-0.158) |

^a^Cumulative values.

Abbreviations: IQR, interquartile range; MAF, mutant allele fraction; na, not available; PD, progressive disease; SD, standard deviation.

**Table S7** Multivariable analysis of p.T790M in plasma and other risk factors for progression.

| **Variable** | **HR** | **95% CI** | ***P* value** |
| --- | --- | --- | --- |
| Age | 1.02 | (1.01–1.02) | *<* 0.001 |
| Man | 1.11 | (0.91–1.35) | 0.310 |
| Smoker | 1.50 | (1.10–2.05) | 0.011 |
| Ex-smoker (1–5 years ago) | 1.42 | (1.05–1.91) | 0.022 |
| Ex-smoker (6–10 years ago) | 0.62 | (0.35–1.12) | 0.112 |
| Ex-smoker (> 10 years ago) | 0.94 | (0.74–1.21) | 0.639 |
| Metastasis (> 1 location) | 1.45 | (1.21–1.73) | *<* 0.001 |
| p.T790M mutation | 2.08 | (1.58–2.74) | *<* 0.001 |

Abbreviations: CI, confidence interval; HR, hazard ratio.

**Table S8** Type of *EGFR* sensitizing mutation detected in baseline plasma, stratified by patient outcome (PFS event).

|  | **Event**  **(n = 53)** | **Event-free**  **(n = 25)** | ***P* value** |
| --- | --- | --- | --- |
| **Mutation type** |  |  |  |
| EG_2235_49D | 16 (30.2%) | 12 (48.0%) | 0.399 |
| EG_2236_50D | 11 (20.8%) | 4 (16.0%) |  |
| EG_2237_55Dt | 2 (3.77%) | 1 (4.00%) |  |
| EG_2239_48Dc | 4 (7.55%) | 0 (0.00%) |  |
| EG_2240_54D | 0 (0.00%) | 1 (4.00%) |  |
| EG_2240_57D | 2 (3.77%) | 0 (0.00%) |  |
| EG_t2573g | 18 (34.0%) | 7 (28.0%) |  |
|  |  |  |  |
| **Exon type** |  |  |  |
| Ex19del | 35 (66.0%) | 18 (72.0%) | 0.790 |
| L858R | 18 (34.0%) | 7 (28.0%) |  |

Abbreviations: *EGFR*, epidermal growth factor receptor; PFS, progression free survival.

**Figure S1** Evolution of MAF in p.T790M-positive patients during the 72-week follow-up period, stratified by patient outcome (PFS event).


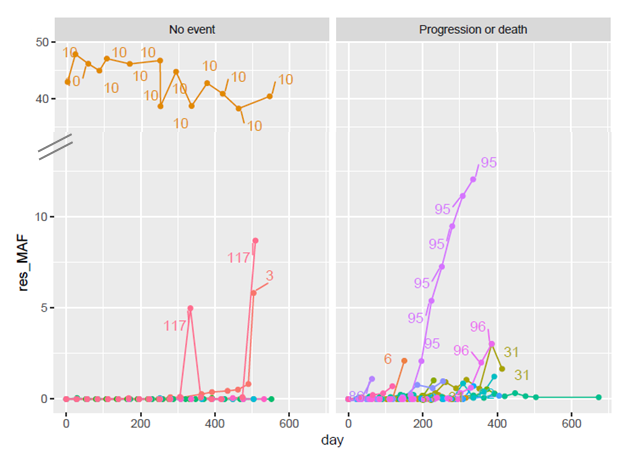


**Figure S2** Description and ratio of sensitizing mutation type detected in plasma samples during the 72-week follow-up.


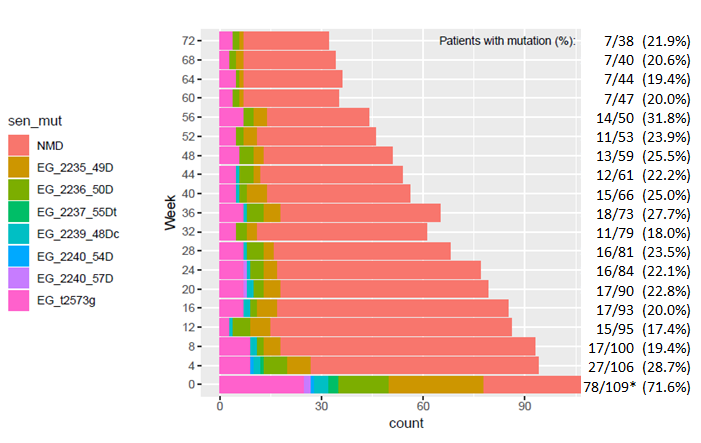


* The patient (ID_88) did not have valid data for all sensitizing mutations.

Abbreviations: NMD, no mutation detected.
